# Schottky model for triboelectric temperature dependence

**DOI:** 10.1038/s41598-018-23666-y

**Published:** 2018-03-28

**Authors:** Martin Olsen, Jonas Örtegren, Renyun Zhang, Salim Reza, Henrik Andersson, Håkan Olin

**Affiliations:** 1 Department of Natural Sciences, Mid Sweden University, Sundsvall, 851 70 Sweden; 2 Department of Electronics Design, Mid Sweden University, Sundsvall, 851 70 Sweden

## Abstract

The triboelectric effect, charging by contact, is the working principle in a device called a triboelectric nanogenerator. They are used as efficient energy transducers in energy harvesting. In such generators the charging of surfaces at contact is followed by a separation of the surfaces increasing the electrical energy which can subsequently be used. Different materials have different triboelectric potentials leading to charging at contact. The temperature dependence of the charging has just recently been studied: the triboelectric effect is decreasing with temperature for a generator of Al-PTFE-Cu. Here, we suggest a mechanism to explain this effect assuming ion transfer using a two-level Schottky model where the two levels corresponds to the two surfaces. The difference in binding energy for ions on the two surfaces then enters the formula for charging. We fit the triboelectric power density as a function of temperature obtained from a two-level Schottky model to measured data for nanogenerators made of Al-PTFE-Cu found in three references. We obtain an average separation energy corresponding to a temperature of 365 K which is of the right magnitude for physically adsorbed atoms. We anticipate that this model could be used for many types of triboelectric nanogenerators.

## Introduction

The triboelectric effect, electrification by contact, has been studied for a long time^1,2^. It has become important in a device called a triboelectric nanoenerator (TENG). They are used as efficient energy transducers in energy harvesting^3–5^. In the generators the charging *Q* of the surfaces with area *A* at contact is followed by a separation *d* of the surfaces increasing the electrical energy which can then be used. The energy is given by

1 $$W=\frac{{Q}^{2}}{2C}=\frac{{Q}^{2}d}{2{\varepsilon }_{0}A}\mathrm{.}$$

Here *ε*_0_ is the permittivity of vacuum. Different materials have different triboelectric potentials leading to charging on contact^6^. Charge transfer can cause strong adhesive forces^7^. The mechanism of charging of metals are well known: the difference in work function between the two metals leads to electron transfer^2,8^.

However for non-metals the charging is partially unknown and its temperature dependence has just recently been studied. Experiments suggest that the charging is done by ion transfer according to McCarty and Whitesides^9^. There are loosely bound atoms on surfaces that can be be transfered to another surface on contact^10^. Triboelectric charging is affected by humidity^11^. The temperature dependence of charging has been studied by Wen *et al*.^12^, Su *et al*.^13^ and Lu *et al*.^14^. The charging energy was found to decrease with increasing temperature for a generator made of Al-PTFE-Cu.

Here, we propose a model to explain this temperature dependence for the Al-PTFE-Cu system assuming charging by ions distributed between the contacting surfaces according to a simple two-level Schottky model. We have fitted our model of the power density or voltage of a triboelectric nanogenerator made of Al-PTFE-Cu to data from Wen *et al*.^12^, Su *et al*.^13^ and Lu *et al*.^14^. We obtained a least square fit for a separation energy corresponding to an average temperature of 365 K. All three fitted values above are in the interval 365 ± 96 K.

## Analysis

First we take a look at the properties of the two-level Schottky model. Then we apply the model to triboelectic charging of triboelectric materials making up a capacitor working as a triboelectric nanogenerator on separation of the capacitor plates.

### Two-level Schottky model

A two-level Schottky system^15^ is a well known model with only two energy levels 1 and 2 separated by the energy *E*. The ratio of the number of particles on the two levels is given by the Boltzmann factor

2 $$\frac{{N}_{2}}{{N}_{1}}={e}^{-E/{k}_{B}T}\mathrm{.}$$

If the number of particles is fixed to *N* = *N*_1_ + *N*_2_ the number of particles on the lower energy level is given by

3 $${N}_{1}=\frac{N}{1+{e}^{-E/{k}_{B}T}}\mathrm{.}$$

We see that at low temperature *T* all the particles are on the lowest level *N*_1_ = *N*, *N*_2_ = 0 and at high temperature they are equally distributed *N*_1_ = *N*_2_ = *N*/2. From the partition function *Z* = 1 + exp(−*E*/*k*_*B*_*T*) for the two-level system many thermodynamical functions of state like internal energy, heat capacity and entropy can be calculated^16^.

### Schottky model applied to triboelectricity

**Figure 1:**
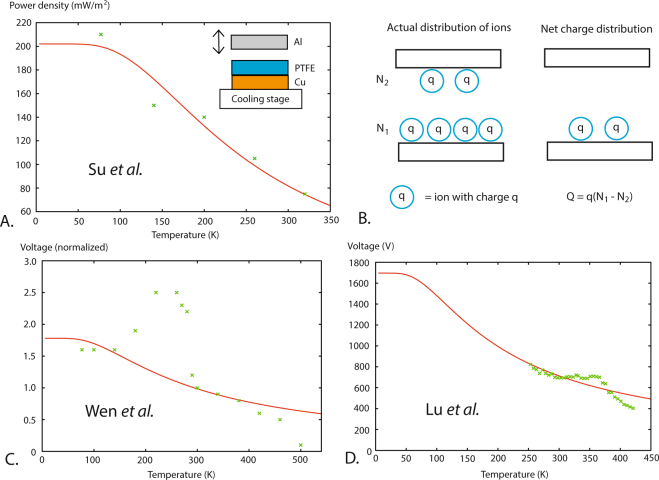
Plots of power density or open circuit voltage as a function of temperature from experiments with triboelectric generators using Al and PTFE as contact materials. (**A**) Plot of the triboelectric power density as a function of temperature using data points from Su *et al*.^13^ fitted to Eq. (6). The curve is fitted to data using the least square method in gnuplot yielding a separation energy corresponding to the temperature *E*/*k*_*B*_ = 451 K. (**B**) Picture of the actual ion distribution and the net charge distribution. (**C**) Plot of normalized voltage as a function of temperature from Wen *et al*.^12^ where Eq. (5) is fitted to data to find *E*/*k*_*B*_ = 374 K. Between 200 K and 300 K Wen *et al*. found a large peak that however was not reproduced by Su *et al*. or by Lu *et al*. shown above. The four highest data points in the peak are excluded from fit. (**D**) Plot of voltage as a function of temperature from Lu *et al*.^14^ where Eq. (5) is fitted to data. We found for this fit *E*/*k*_*B*_ = 269 K.

When two materials with a difference *E* in binding energy for ions are brought into contact a two levels system will develop. We assume that the materials have *N* ions in common that can be distributed between them. The net charge of the capacitor is then *Q* = *q*(*N*_1_ − *N*_2_), see Fig. 1B. We obtain

4 $$Q=qN(\frac{2}{1+{e}^{-E/{k}_{B}T}}-1)\mathrm{.}$$

The capacitor voltage is given by *U* = *Q*/*C* where *C* = *ε*_0_*A*/*d* is the capacitance so

5 $$U=\frac{qN}{C}(\frac{2}{1+{e}^{-E/{k}_{B}T}}-1)\mathrm{.}$$

The energy of a capacitor is *W* = *Q*^2^/(2*C*), see Eq. (1). The triboelectric power density *P* = *Wf*/*A* then becomes

6 $$P=\frac{{N}^{2}{q}^{2}f}{2CA}{(\frac{2}{1+{e}^{-E/{k}_{B}T}}-1)}^{2},$$

where *f* is the charging/discharging frequency. The power density *P* is thus proportional to the frequency while the maximum output power from the device to the load resistance *R* given by *U*^2^/*R* is independent of the frequency. For low temperature Eq. (6) becomes *P* ≈ *N*^2^*q*^2^*f*/(2*CA*) independent of temperature and for high temperature *T* Eq. (6) reduces to

7 $$P\approx \frac{{N}^{2}{q}^{2}f}{2CA}{(\frac{E}{2{k}_{B}T})}^{2}\mathrm{.}$$

Using the least square method in gnuplot we fitted the data points in Fig. 1A,C and D to the Schottky model, see Table 1. The three fitted values of *E*/*k*_*B*_ are all within the interval *E*/*k*_*B*_ = 365 ± 96 K. This temperature corresponds to the energy 31 ± 8 meV. Is this value of *E* consistent with an ion Schottky model? What kind of ions which are exchanged between Al and PTFE in the triboelectric generator experiments is unknown but a comparison is made in Table 2 for some adsorbed atoms that could work as a model to estimate the separation energy. In this table are shown work function differences between covered and uncovered surfaces for three kind of noble gas atoms adsorbed to three different noble metals. We see that for example the difference in work function for an Ar atom when it sits on an Au surface compared to when it sits on an Ag surface is 20 meV. This energy should correspond to the separation energy *E*. The energy difference between different substrates for an adsorbed atom seems then to be of the right magnitude to support an ion Schottky model.

**Table 1:** Power density as a function of temperature from three references measuring on triboelectric nanogenerators using Al and PTFE as contact materials. Su *et al*. and Lu *et al*. used the frequencies 0.05 Hz and 3 Hz respectively. For Wen *et al*. two experimental devices were used: one for low temperature regime 77 K–300 K and one for high temperatures 300 K–500 K. The low temperature device used the frequency 0.03 Hz and the high temperature device used the frequency 0.3 Hz. The average of the fitted characteristic temperatures is *E/kB* = 365 K. All fitted values are within the interval *E/kB* = 365 ± 96 K.

Reference	Temperature interval	Fitted *E*/*kB*	Power density v.s. temperature
Wen *et al*.^12^	77 K–500 K	374 K^1^	Decreasing trend but peak between 200 K and 300 K.
Su *et al*.^13^	77 K–320 K	451 K^2^	Decreasing with temperature, see Fig. 1A.
Lu *et al*.^14^	250 K–420 K	269 K^3^	Decreasing trend but plateau between 290 K and 370 K.
Average		365 K^4^	

Note 1: Model fitted to 11 out of 15 data points to calculate *E/kB*, see Fig. 1C. Four data points at peak excluded from fit.

Note 2: Model fitted to all five data points, see Fig. 1A.

Note 3: Model fitted to all 35 data points, see Fig. 1D.

Note 4: All fitted values from Wen *et al*., Su *et al*. and Lu *et al*. are in the interval *E*/*k*_*B*_ = 365 ± 96 K.

**Table 2:** Average of measured work function differences for Ar, Kr and Xe atoms adsorbed to substrates of Au, Ag and Cu from Hückstädt *et al*.^23^. The work function difference is for a mono layer of adsorbate compared to a clean substrate surface.

	Ar	Kr	Xe
Au(111) substrate	0.38 ± 0.03 eV	0.42 ± 0.02 eV	0.53 ± 0.03 eV
Ag(111) substrate	0.40 ± 0.03 eV	0.46 ± 0.02 eV	0.59 ± 0.03 eV
Cu(111) substrate	0.42 ± 0.03 eV	0.49 ± 0.03 eV	0.62 ± 0.03 eV

How is adsorption affected by temperature? The Langmuir adsorption isotherm model is considering the adsorption of gas atoms on to a surface. This could be applied before surface contact. Neglecting interactions between the adsorbed atoms, for surface coverage 0 < *θ* < 1 the Langmuir model can be used as an approximation. We have atoms in a gas with concentration [*A*] over a surface with available sites at a concentration [*S*]. We have then the equilibrium reaction $A+S\rightleftarrows AS$ where the adsorbed state is denoted *AS*. A temperature dependent equilibrium constant *K*(*T*) can then be written using the law of mass action^17^

8 $$K(T)=\frac{[AS]}{[A][S]}\mathrm{.}$$

The occupied surface sites concentration [*AS*] will be proportional to the surface coverage *θ* while [*S*] will be proportional to the number of vacant sites 1 − *θ*. The gas concentration [*A*] is denoted *c*. We obtain

9 $$K(T)=\frac{\theta }{\mathrm{(1}-\theta )c},$$

or

10 $$\theta =\frac{K(T)c}{1+K(T)c}\mathrm{.}$$

The equilibrium constant *K*(*T*) is found to be decreasing with increasing temperature. This implies reduced surface coverage *θ* at higher temperatures. We see also that as the concentration *c* increases, the surface coverage *θ* approaches unity.

However, in our triboelectric system we have two surfaces in temporary contact and most of the ion transfer probably happens when almost no gas is in between the surfaces so a better model of ion transfer should be that of surface diffusion^18^. Atoms jumps on the surfaces and probably even between them at contact. The jump frequency Γ on a surface is given by^19^

11 $${\rm{\Gamma }}\approx \frac{{k}_{B}T}{h}{e}^{-{E}_{A}/{k}_{B}T},$$

where *k*_*B*_*T*/*h* is the thermal vibration frequency of the lattice, *h* is the Planck constant and *E*_*A*_ is the activation energy to escape from the potential well. The higher temperature *T* the larger jumping frequency Γ. Higher temperature thus increase the ion transfer rate between the surfaces but should not affect the equilibrium distribution of ions on the two surfaces.

## Discussion

Because the mechanism causing the temperature dependence for voltage and power density of a triboelectric generator is still unknown, we have here suggested a simple Schottky model. This model seems to be able to explain the main features found in experiments by Wen *et al*., Su *et al*. and Lu *et al*.: decreasing voltage and power density with increasing temperature with a characteristic temperature of *E*/*k*_*B*_ = 365 ± 96 K. In Wen *et al*. the general trend is also decreasing voltage with temperature. However, the experimentally found large peak in capacitor voltage between 200 K and 300 K can not be explained within our Schottky model, see Fig. 1C. However, the peak is not reproduced by Su *et al*. for the same temperature interval. Lu *et al*. studied the temperature interval 250 K–420 K, which covers half the interval where Wen *et al*. found the peak, but no peak was found in the experiment by Lu *et al*.

How does our Schottky model compare to other ion transfer models found in the literature? McCarty and Whitesides^9^ are using a model where the charge of the ions on a dielectric plate is neutralized by bound counter ions close to the surface of this material. The ions thus “belong” to this plate making it neutral. When another plate is brought in contact some of the ions get transferred to the other plate. The ion transfer is then creating an electric field between the plates which counteract more ion transfer. In this way the number of ions on the two surfaces can be calculated and hence the net charge on the plates. In our model we do not assume that the ions belong to one plate: we are assuming that they come from both plates. They are then distributed between the plates depending on the difference in work function for ions for the two plates creating a charge difference between the electrodes. However, we can include the effect of the presence of bound net charges on one plate into our model by adding a constant charge *Q*_*B*_ to Eq. (4):

12 $$Q=qN(\frac{2}{1+{e}^{-E/{k}_{B}T}}-1)+{Q}_{B}\mathrm{.}$$

The voltage then becomes *U* = *Q*/*C* as before. However, this will only add a constant to the voltage with the same type of temperature dependence because the bound charge *Q*_*B*_ will be temperature independent. The expression for the power density *P* will be more complicated because it contains the charge *Q* squared. For simplicity we assume *Q*_*B*_ = 0.

How valid is our model in view of that the particles are charged and thus change the energy difference between the levels as the number of ions changes? The energy difference *E* is constant only for small net charge *Q* because of the electrostatic energy induced by the separation of charges. This effect tends to increase the effective *E* making it harder to transfer more charges. The electric field between the plates are given by *Q*/(*ε*_0_*A*) where *A* is the plate area and *ε*_0_ is the permittivity of vacuum. The force on one charge carrier is then *qQ*/(*ε*_0_*A*) so the work required to transfer one particle the distance *d* between the plates becomes *qQd*/(*ε*_0_*A*), see McCarty and Whitesides^9^. The energy difference *E* should then be corrected to *E* + *qQd*/(*ε*_0_*A*). However, before the plate separation the plate distance *d* is small and the correction term should be possible to neglect. After the separation, as *d* increases further, the ions would quickly get trapped on the surfaces they sit on^9^. The approximation of a constant *E* should then be acceptable.

How is the operating frequency of the triboelectric generator affecting the Schottky model? The frequency of charge/discharge of the triboelectric generators could affect the ratio of the number of ions on the two surfaces because the Schottky model assumes thermal equilibrium, and if the frequency is too high the system might not have time to equilibrate. The experiments of Wen *et al*., Su *et al*. and Lu *et al*. uses frequencies ranging from 0.03 Hz to 3 Hz, see legend of Table 1. These are rather low frequencies and no features in the curves seems to respond to this effect. However, a future application of triboelectric generators to high frequencies could change this.

Can the Schottky model be applied to all nanogenerators containing non-metals? No. For a generator with one sheet covered with TiO _2_-nanotubes the reverse temperature behavior was found with increasing charging with temperature^13^. This latter system involves however nanomaterials which may be similar to the materials in gecko feet with its long hair yielding a large effective contact area^20,21^. For gecko feet complex behavior of contact force on temperature and humidity has been found^22^. This may indicate the need for a more complex model for this latter system so that a simple Schottky model would not suffice. For a system not including nanomaterials the Schottky model should however work.

The Schottky model seems to work for a triboelectric nanogenerator made of Al-PTFE-Cu. We anticipate that the Schottky system could be used as a model of many types of normal triboelectric nanogenerators containing non-metals.
